# A Focused Multiple Reaction Monitoring (MRM) Quantitative Method for Bioactive Grapevine Stilbenes by Ultra-High-Performance Liquid Chromatography Coupled to Triple-Quadrupole Mass Spectrometry (UHPLC-QqQ)

**DOI:** 10.3390/molecules22030418

**Published:** 2017-03-07

**Authors:** Elías Hurtado-Gaitán, Susana Sellés-Marchart, Ascensión Martínez-Márquez, Antonio Samper-Herrero, Roque Bru-Martínez

**Affiliations:** 1 Departamento Agroquímica y Bioquímica, Facultad de Ciencias, Universidad de Alicante, 03690 Alicante, Spain; ehurtadog7@gmail.com (E.H.-G.); susana.selles@ua.es (S.S.-M.); asun.martinez@ua.es (A.M.-M.); antonio.samper@ua.es (A.S.-H.); 2 Genomics and Proteomics Unit, SSTTI Universidad de Alicante, 03690 Alicante, Spain; 3 Instituto de Investigación Sanitaria y Biomédica de Alicante ISABIAL-Fundación para el Fomento de la Investigación Sanitaria y Biomédica de la Comunitat Valenciana FISABIO, 03010 Alicante, Spain

**Keywords:** UHPLC, MRM, resveratrol, piceid, piceatannol, epsilon-viniferin, pterostilbene, cell culture, bioconversion, wine

## Abstract

Grapevine stilbenes are a family of polyphenols which derive from *trans*-resveratrol having antifungal and antimicrobial properties, thus being considered as phytoalexins. In addition to their diverse bioactive properties in animal models, they highlight a strong potential in human health maintenance and promotion. Due to this relevance, highly-specific qualitative and quantitative methods of analysis are necessary to accurately analyze stilbenes in different matrices derived from grapevine. Here, we developed a rapid, sensitive, and specific analysis method using ultra-high-performance liquid chromatography coupled to triple-quadrupole mass spectrometry (UHPLC-QqQ) in MRM mode to detect and quantify five grapevine stilbenes, *trans*-resveratrol, *trans*-piceid, *trans*-piceatannol, *trans*-pterostilbene, and *trans*-ε-viniferin, whose interest in relation to human health is continuously growing. The method was optimized to minimize in-source fragmentation of piceid and to avoid co-elution of *cis*-piceid and *trans*-resveratrol, as both are detected with resveratrol transitions. The applicability of the developed method of stilbene analysis was tested successfully in different complex matrices including cellular extracts of *Vitis vinifera* cell cultures, reaction media of biotransformation assays, and red wine.

## 1. Introduction

**Figure 1:**
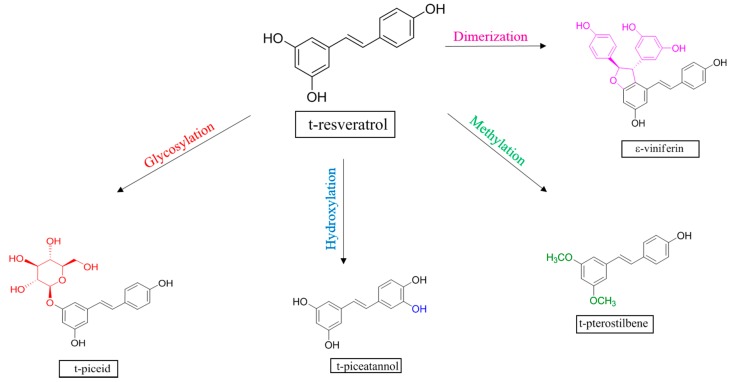
Grapevine stilbenes: t-Resveratrol and derivatives produced by different biochemical reactions.

Grapevine (*Vitis vinifera* L.) stilbenes are a family of polyphenols which derive from *trans*-Resveratrol (*trans*-R: 3,5,4′-trihydroxystilbene), found in different plant organs, including berries and, consequently, also in wines. *Trans*-R is synthesized as an end-product of a branch of the phenylpropanoid pathway; further *trans*-R may undergo biochemical modifications, such as hydroxylation, glycosylation, methylation, or oligomerization, giving rise to several bioactive derivatives (Figure 1). The synthesis and accumulation of stilbenes is considered to be part of the general defense mechanism as these are considered as the phytoalexins of *Vitis* species since they display strong antifungal and antimicrobial activities [1,2,3]. In fact, *trans*-R is accumulated in grapevine vegetative tissues and berries, as well as in cell cultures in response to abiotic and biotic stress [1,4,5,6,7,8,9]. Stilbenes have been demonstrated to have a large number of bioactive properties in relation to major human diseases such as cancer, atherosclerosis, or neurodegeneration [10]. Many studies have reported the potential of *trans*-R in human health as an antioxidant in model systems of cardiovascular diseases [11], as a cytotoxic agent for several cancer lines [12,13,14,15], as an anti-inflammatory, anti-aging, and antioxidant agent [16,17], having a beneficial role on neurological system [18], as well as health maintenance and survival in adverse scenarios related with obesity and aging [19,20]. Moreover, other studies have demonstrated that some *trans*-R polyhydroxy and polymethoxy derivatives also exhibit enhanced pharmacological activity and bioavailability [21]. Such potential of *trans*-R and other stilbenes have called the attention of numerous companies producing a large variety of dietary supplements and cosmetics. Stilbenes are presently in natural foods, such as grapes, berries of *Vacinum* species, cocoa, and peanuts, and their derived products [22,23,24,25,26]. In this sense, stilbenes, especially resveratrol, play a key role in wine health-promoting properties (see [27] for a review). Once the potential health benefits of stilbenes have been demonstrated, it is necessary to have highly specific qualitative and quantitative methods to accurately analyze stilbenes in different matrices derived from grapevine.

Stilbenes have been detected by different analytical techniques coupled to chromatographic separation. In wine and grape samples, several research groups report the separation and detection of phenolic compounds using HPLC coupled to detection by either UV-VIS and fluorescence [28], or MS and MS/MS [29,30,31,32,33]. However, the specificity of detection of these techniques is limited because different compounds, having similar mass or spectroscopic properties may co-elute in a chromatographic run. The incorporation of triple quadrupole mass spectrometers (HPLC-QqQ) to analyze phenolic compounds significantly improved the detection and allowed the simultaneous quantitation of multiple compounds in complex matrices in a reduced time. Jaitz et al. quantified 11 polyphenolic compounds from red wines in less than 13 min [34]. In other plant matrices, HPLC-QqQ MS was applied to quantify 28 polyphenols in cocoa extracts [35] and, more recently, 20 polyphenols from carob (*Ceratonia siliqua* L.) flour by improving the chromatography separation and speed by using ultra-high-performance liquid chromatography (UHPLC) [36]. UHPLC allows faster separation and enhanced chromatographic resolution as compared to classical HPLC, due to reduced particle size and increased column pressure (up 1000 bar). UHPLC coupled to triple quadrupole mass spectrometry using multiple reaction monitoring (MRM) mode has been proposed as an alternative for fast analysis of phenolic compounds in grapes and wine. UHPLC-QqQ in MRM mode has been used to detect and quantify 152 polyphenols in rose wine, in 30 min [37], which is quite a noticeable improvement for the analysis of general wine phenolics. The large increase in the number of compounds analyzed is achieved at expense of longer chromatographic runs and possibly, some loss of specificity. and/or sensitivity when different co-eluting compounds share transitions or are present at dramatically different abundances.

Thus, when focusing on particular compound families (i.e., stilbenes), improved methods that provide effective compound separation and quantitation in different kinds of matrices in short chromatographic runs are necessary.

Our study aimed at developing an analytical method for efficient separation and accurate quantification of stilbenes produced by *Vitis vinifera*, of application to different matrices, such as red wine and plant material extracts, among others. Five important bioactive stilbenes were chosen for analysis by UHPLC-QqQ MS operated in MRM detection mode, in order to improve the detection and quantitation of stilbenes in a short time of analysis.

## 2. Results

### 2.1. Optimization of Ion Source Parameters

To carry out this work we utilized an Agilent 6490 QQQ instrument equipped with a JetStream^®^ ion source. As this instrument uses a fixed 380 V fragmentor voltage this parameter cannot be optimized. Glycosylated polyphenols, such as piceid, are prone to in-source fragmentation, thus, it was necessary to optimize some parameters of the ion source to minimize this effect. To that, a standard solution of piceid was directly infused into the instrument and several parameters were tested to maximize the signal intensity. The cell acceleration voltage, tested at 2 and 5 V, was set at 2 V for subsequent analysis. Next, the gas curtain temperature was tested at five values in the range of 250 °C to 400 °C, with 300 °C being selected as a compromise between signal intensity and compound in-source fragmentation. The gas flow had almost no effect on fragmentation, but a clear improvement in the signal intensity was observed at 12 L/min. Eventually, we tested several capillary voltages in a range of 2500–4500 V, getting the best result at 3000 V. As the other stilbene compounds do not undergo in-source fragmentation, the source parameters optimized for piceid were used throughout in subsequent analysis.

### 2.2. Selection of MRM Transitions and Optimization of Chromatographic Separation

Five stilbenes found in *Vitis* species, namely t-Piceid, t-Piceatannol, t-R, t-ε-Viniferin and t-Pterostilbene were used in this study. MRM conditions were first optimized in positive detection mode. Each compound was individually infused and, while operating the MS instrument in product ion scan mode, four discrete collision energies were applied (0, 10, 20, and 30 eV) in order to detect fragment ions and automatically select the four most intense ones. Then, the collision energy for each transition was optimized by applying a 0–40 eV ramp. The transitions selected for each compound, optimal collision energies and intensities are listed in Table 1. Since the transitions selected for piceid gave rise to non-specific peaks in the chromatogram, the fragment ions were changed for those of *trans*-R, thus producing clean chromatographic traces. Only the product ion *m*/*z* 229.1 was kept as being the most abundant transition for piceid. Due to in-source fragmentation, piceid generates resveratrol parent ions that are detected through the resveratrol transitions; thus, peak overlapping of resveratrol and piceid must be avoided.

**Figure 2:**
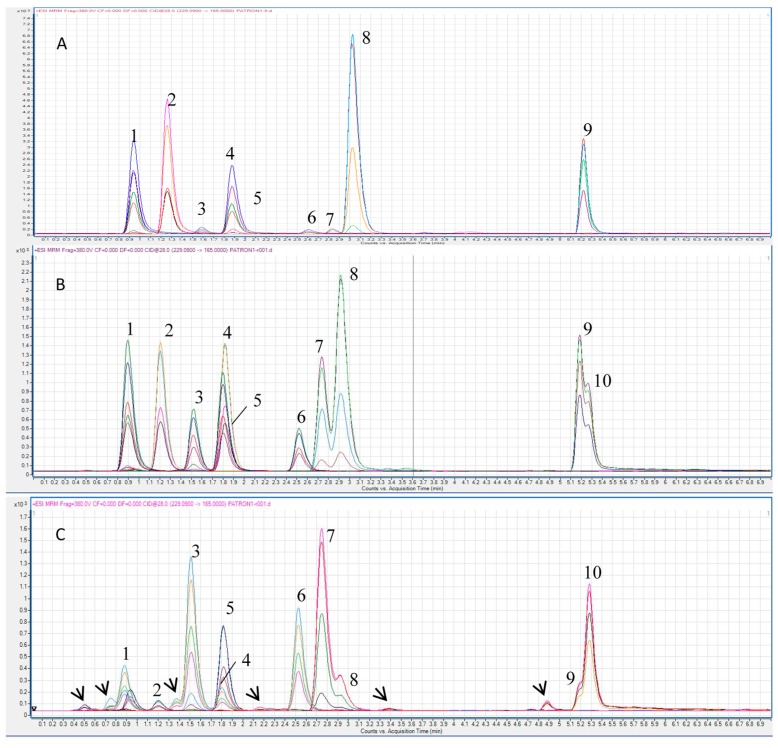
Chromatogram of MRM transitions of stilbenes in the standard mix. 1: *trans*-Piced; 2: *trans*-piceatannol; 3: *cis*-Piceid; 4: *trans*-Resveratrol; 5: *cis*-Piceatannol; 6: *cis*-Resveratrol; 7: *cis*-Viniferin; 8: *trans*-Viniferin; 9: *trans*-Pterostilbene; 10: *cis*-Pterostilbene. (**A**): Without UV light exposition; (**B**) short exposition to UV light; and (**C**) prolonged exposition to UV light. Arrows indicate the presence of new compounds originated by the prolonged UV exposition.

To optimize the separation conditions of these five stilbenes in a short chromatographic run, a mix of standard was injected in a UHPLC-QQQ from Agilent (Santa Clara, CA, USA). Several trials were conducted with different gradients in the mobile phase, containing water as solvent A and acetonitrile or acetonitrile:methanol as solvent B. Using several binary gradients starting at 5% acetonitrile resulted in a poor resolution and late elution of peaks and, in addition, peaks corresponding to *cis*-Piceid and *trans*-R appeared overlapping. To improve the poor separation observed, ternary gradients of water:acetonitrile:methanol were assayed starting at 35% B for an earlier peak elution. A 10 min gradient was tested using from 40:60 up to 70:30 acetonitrile:methanol as solvent B, thus achieving a significant improvement in the chromatographic separation, 70:30 being the proportion that showed the best separation of all the stilbenes. A key improvement of this chromatographic optimization has been the *cis*-Piceid peak neat separation from the *trans*-R peak, otherwise *trans*-R quantification would be overestimated as the sum of the signal of both compounds. As can be seen in Figure 2, the gradient was finally shortened to 6 min (the total run after equilibration was 8 min) without compromising resolution. Minor peaks, supposed to be the respective *cis*-isomers as judged by the co-elution of all compound-specific transitions, were also detected. As mainly the *trans* isomers are commercialized as standards, we exposed the standard mixture to UV light to promote photoisomerization in order to characterize the peaks corresponding to the *cis* isomers. Under the optimized chromatographic conditions, all stilbenes both, as *trans* and *cis* isomers were detectable and eluted clearly differentiated (Figure 2). The only co-eluting compounds were *trans*-R and *cis*-piceatannol, but such overlapping is unambiguously resolved by their specific transitions.

### 2.3. MRM Quantitation of Stilbenes in a Standard Mixture

After MRM has been successfully optimized to detect target stilbenes produced by *V. vinifera,* performance of the method to quantify these compounds was assessed. For quantitative MRM, the quantifier transition chromatogram of each targeted precursor *m*/*z* was used, and the rest of transitions provided the evidence of the presence or absence of the stilbene in the standard mixture.

The quantifier transitions used for each compound were *m*/*z* 391.1→229.1 for piceid, *m*/*z* 229.09→107.1 for resveratrol, *m*/*z* 245.08→135.1 for piceatannol, *m*/*z* 455.15→107.1 for viniferin, and *m*/*z* 257.12→133.1 for pterostilbene. Detailed data of metabolite fragmentation is provided as Supplementary Material in file S1. The optimized transition for each specific compound constitute a robust assay that can be used to quantify the target stilbenes in complex mixtures by MRM analysis. The estimated concentration of standard stilbene solutions were calculated, results are shown in Supplementary Material file S1.

In order to explore the linear dynamic range of the instrument’s response and the limits of detection (LOD) and quantification (LOQ) for each stilbene, serial dilutions of the standard mixture covering several orders of magnitude were analyzed with the MRM method and results are summarized in Table 1 and Tables S1–S5 in Supplementary Material, file S2. Linear relationship between peak area and concentration was calculated for all the stilbenes, considering only the peaks corresponding to *trans* isomers. At high concentrations of stilbenoids a loss of linearity was apparent, holding linearity up to 40 mg/L for piceid and pterostilbene, 50 mg/L for Resveratrol and 160 mg/L for piceatannol and ε-viniferin. The response of the detector was linear over ca. three orders of magnitude on average for the set of stilbenes analyzed. To determine LOD and LOQ the signal-to-noise ratio was used establishing rather conservative cut-off values. The LOD ranged from 0.04 to 0.12 mg/L and the LOQ from 0.07 to 0.22 mg/L.

### 2.4. Examples of Applications of the Optimized Method

**Figure 3:**
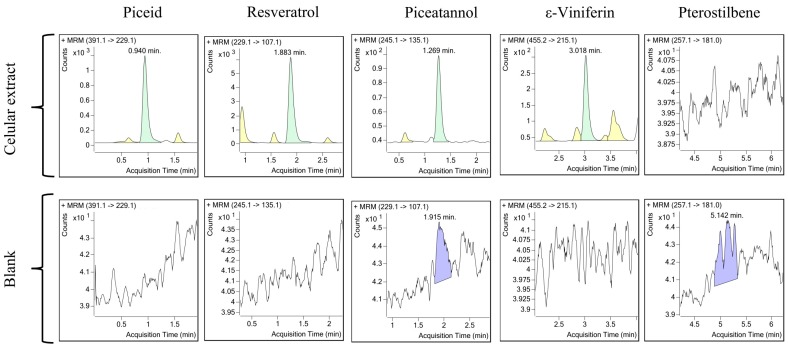
Quantitation of stilbenes from *V. vinifera* cell cultures upon elicitation. Transition chromatograms for each compound are shown. Blank is methanol, 80%.

In order to validate the applicability of the MRM method developed we carried out stilbene analysis in different experimental scenarios. On the one hand, we applied the method to the analysis of stilbenes extracted from grapevine cell cultures. *V. vinifera* cell cultures might produce some of these five stilbenes incubated either in normal growth medium or under elicitation conditions [38]. To that, several samples of methanolic extracts of elicited cell cultures of *V. vinifera* were used for the applications of the method in complex mixtures. Use of external calibration curves with three levels of standards enable the detection and quantification of all stilbenes assayed with the exception of pterostilbene, which was absent in this sample. The quantifier transition chromatogram for each stilbene detected is shown in Figure 3 and the calculated concentrations are in Table 2. As expected, *trans*-R was the most abundant stilbene produced by *V. vinifera* under elicitation, but others including piceid, piceatannol, and ε-viniferin were also present at significant levels.

**Figure 4:**
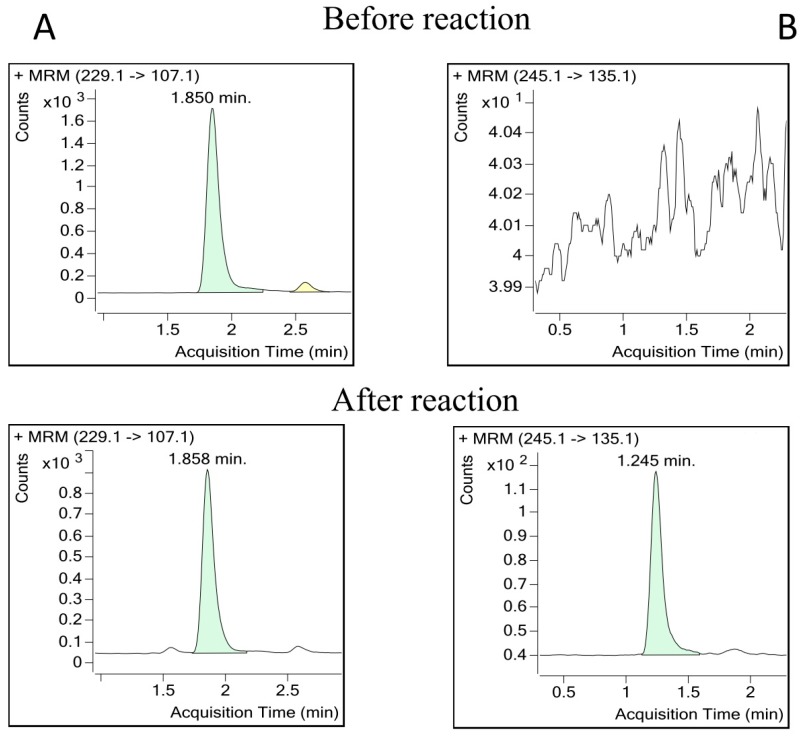
Bioconversion of *trans*-Resveratrol to *trans*-Piceatannol. (**A**) Resveratrol transition chromatogram; and (**B**) piceatannol transition chromatogram.

On the other hand, the method was applied to follow the enzymatic assay of bioconversion of *trans*-R through an hydroxylation reaction into a tetrahydroxystilbene [39]. A solution of *trans*-R standard and NADPH substrates was incubated in the presence of a protein crude extract from grapevine cell cultures. Stilbene reaction products after one hour incubation, as well as residual *trans*-R substrate, was extracted and analyzed with the developed method. As shown in Figure 4A, *trans*-R peak decreased in abundance at the time a new peak at the same retention time as *trans*-Piceatannol appeared, thus confirming that grapevine cell extracts have resveratrol hydroxylation activity.

**Figure 5:**
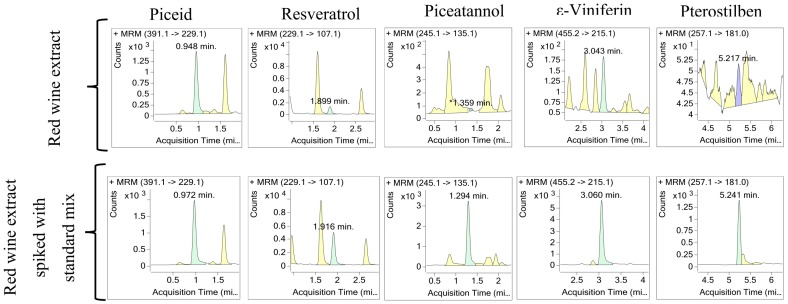
Quantitation of stilbenes in red wine extract and in the same red wine extract spiked with a standard stilbene mixture. Transition chromatograms for each compound are shown.

Eventually, red wine samples were analyzed using the MRM method in order to detect and quantify the absolute amount of the five targeted stilbenes in this highly complex matrix. To reduce interferences and increase sensitivity of the assay, stilbenes from both spiked and non-spiked samples were recovered by solid phase extraction [40]. However, the resulting sample is still highly complex as evidenced by an HPLC-UV analysis (Figure S2) in conditions described by Martinez-Esteso et al. [5]. As shown in Figure 5, out of the five stilbenes analyzed, piceid, resveratrol and ε-viniferin were present, but only their respective *trans* isomers were quantified (Table 3). In addition, their *cis* isomers as well as other viniferins were also present and, presumably, in higher amounts as judged by the intensity of their corresponding transition peaks, but the lack of standards precluded their absolute quantification.

Although the quantitation limits of the stilbenes in this method can be above the existing amount in wines, the protocol used for sample preparation combining solid phase extraction, leading to 33-fold concentration, and several calibration points of standard spiking allows for the confident analysis of concentrations in wine well below LOQ, as is the case for ε-viniferin.

## 3. Discussion

In the context of the occurrence of bioactive stilbenes in natural sources, a liquid chromatography-based effective methodology to analyze both qualitatively and quantitatively the content of these compounds in different matrices is necessary. The way towards efficient analytical methods of polyphenols has been focused in making both a more efficient chromatographic separation and a more specific detection system. Thus, we have currently arrived to UHPLC, that lead to sharp peak resolution in short runs, and mass detectors, from which the triple quadrupole (QqQ) operated in SRM/MRM mode offers the highest specificity, excellent sensitivity and an extreme multiplexing capacity. Overall benefits involve a significant reduction of the analysis time and, thus, of costs. Using HPLC-MS/MS stilbenes from elicited grapevine cell cultures were qualitatively analyzed, detecting up to 20 different compounds in ca. 30 min chromatographic runs [33]. There also currently exist UHPLC-MRM methods focused on stilbenes [41], however these were specifically designed to analyze resveratrol and piceid metabolites in urine bearing negatively-charged groups such as glucuronides and sulfates derived from phase II reactions. Recent examples of the application of UHPLC-QqQ to the analysis of polyphenols in specific matrices, as rose wine [37] or several fruits and beverages [42], have extensively demonstrated these capacities. Trying to exploit these capacities to extreme has led to develop the simultaneous assay of 152 different polyphenols in non-processed samples of rose wine [37], but this remarkable achievement was done at the cost of longer time of analysis, introduction of quantitative uncertainty and loss of specificity for some compounds which include stilbenes. In particular, the *trans* and *cis* configurations of resveratrol, piceid and two viniferin isomers of unknown configuration included in such analysis eluted between 12 and 18 min. The short availability of polyphenolic standards and, in particular, most of the *cis* isomers of stilbenes, precludes the direct quantification of these configurations. The similarity between *cis* and *trans* fragmentation spectrum allows their easy detection with the major transitions of the *trans* standards, as seen in UV-exposed standard mix (Figure 2), but the different relative abundance of fragment ions introduces uncertainty in their absolute quantitation. By means of an extensive UV exposition we intended to quantitatively generate the corresponding *cis* standards but the appearance of new peaks from almost every compound revealed that such *trans*/*cis* photoisomerization is not quantitative as the reaction may proceed to generate other isomers [43], so we have not performed *cis*-isomer quantitation. The same is true for the diversity of viniferins resulting from the coupling of two resveratrol units producing several dehydrodimers [44] that, in turn, may also have *trans* and *cis* configurations. In-source fragmentation is frequently occurring in glucoside and galactoside derivatives of phenolic compounds as syringetin [42] or piceid seen here. This phenomenon lead to detect aglycone at the retention time of the glycosylated form, in addition to the aglycone at its own retention time. Although we tried to avoid this phenomenon through source parameters optimization, only an improvement of the piceid signal was achieved, perhaps because a key parameter of the ion source to attain such goal, i.e., the fragmentor voltage [45], is fixed for this instrument. For MRM detection, co-elution of analytes is not generally a problem unless these are detected by the same transitions, as is the case for the glycosylated *cis*-Piceid and the aglycone *trans*-Resveratrol due to the in-source fragmentation of the former, therefore co-elution has to be avoided. Quantitation of *trans*-Resveratrol, which has been extensively performed in plant extracts, juices and especially wines during the last two decades for its relevance as bioactive compound [46], might therefore be deeply affected by this issue. Although these compounds co-eluted in most of the chromatographic conditions assayed here, we were eventually able to establish the right conditions for a neat separation, becoming the first UHPLC-MRM method to solve this problem, thus assigning each transition peak area to a unique stilbene compound. The use of methanol as solvent in the mobile phase has been reported to increase considerably the response when the instrument is operating in positive ionization mode (ESI+) [47]. In the most of methods to determine and quantify polyphenols based on LC-MS or MS/MS [34,37,42,48,49,50], solvent B consisted of acetonitrile or methanol, but here we achieved the best chromatographic resolution using a mixture 70:30 acetonitrile:methanol as solvent B. 

As mentioned above, resveratrol dimers exist in diverse isomeric forms but only the ε-*trans*-viniferin is commercialized as standard. The *cis* isomer was produced by UV exposition and, conversely to the rest of *cis* isomers for the stilbenes included in this study, it eluted earlier than the *trans* (Figure 2). This has some implications on the tentative assignment of structural variants of these compounds in complex samples i.e., wine, when no standards are available. In previous studies using HPLC coupled to an ion trap mass spectrometer we found several viniferin dimers eluting between resveratrol and pterostilbene standards [5,32], thus, the gap between ε-*trans*-viniferin and pterostilbene in the current method is not worthy to shorten as complex samples may contain these isomers, as can be seen in Figure 5.

MRM has proven to be a successful technique for detection and quantitation of target metabolites. The set of transitions selected for each stilbene show a high specificity, and allow both their determination and quantitation in an efficient manner in the standard mixture. Although we monitored all of them only the most intense transition was used as the quantifier, and the peak area of it and the nominal concentration was used to construct the calibration curves for each compound. The limit of quantitation (LOQ) and limit of detection (LOD) were based on the S/N of standards and the values, in the tens of µg/L range, were well below the levels typically described for these compounds, above the hundreds of µg/L range. Only pterostilbene can be a scarcer compound in biological samples as its accumulation in grapevine tissues occurs locally and in response to pathogen infection [51], and even in engineered cell lines its cytotoxicity and fast degradation precludes its high accumulation [38]. The response of the detector was linear over ca. three orders of magnitude, thus covering the levels found in the majority of matrices. In this way, target stilbenes could be quantitatively analyzed in different types of samples, including grapevine cell extracts, bioconversion reaction media, and wine.

In conclusion, major achievements of the analytical method developed include a higher analysis throughput by means of a significant shortage of chromatographic runs and a higher specificity and accuracy provided by a good separation of compounds detected with the same transitions. Focusing on the particular family of stilbene compounds provides the analytical advantages over broad compound coverage methods. Many studies were done in the past twenty years on resveratrol and piceid content in natural sources, such as fruits, juices, and beverages, including wine, as well as cultures of grapevine cells. There is a renewed interest in other stilbenes such as piceatannol and pterostilbene, which show enhanced pharmacological and pharmacodynamical properties with respect to its relative resveratrol and, oligomers of resveratrol begin to being explored as neuroprotecting compounds [52]. The number of compounds quantifiable with this method may increase as new stilbenes become available as standards.

## 4. Materials and Methods

### 4.1. MRM Method Optimization

#### 4.1.1. Chemicals and Reagents

*Trans*-Resveratrol, *trans*-Piceid, and *trans*-Pterostilbene standards were supplied by Chromadex (Irvine, CA, USA); *trans*-ε-Viniferin from Actichem (Mountaban, France) and *trans*-Piceatannol were from Cayman Chemical Company (Ann Arbor, MI, USA). Thermo Fisher scientific (Waltham, MA, USA) supplied formic acid, acetonitrile, water, methanol, and trifluoroacetic acid of LC-MS grade.

#### 4.1.2. Preparation of Standard Solutions

Stock solutions of each individual standard stilbene were prepared in 80% methanol, resveratrol (500 mg/L), piceid (400 mg/mL), piceatannol (160 mg/mL), ε-viniferin (800 mg/mL), and pterostilbene (400 mg/mL). These stock solutions were used to prepare a mixture containing the following concentration of standards: resveratrol (50 mg/L), piceid (40 mg/mL), piceatannol (90 mg/mL), ε-viniferin (90 mg/mL), and pterostilbene (40 mg/mL). Dilutions of either pure of mixed standards were carried out in 80% methanol. To generate *cis*-isomers of the standards, the stilbene mixture was exposed to UV light using a germicide lamp for 3 h (short exposition) or 24 h (prolonged exposition).

#### 4.1.3. Chromatography and Mass Conditions

LC-MS analysis were performed in an Agilent 1290 Infinity UHPLC coupled to an Agilent 6490 triple quadrupole mass spectrometer through an Agilent Jet Stream^®^ ion source (Agilent, Santa Clara, CA, USA). Separation of analytes was performed on a Zorbax Extended C18 column (2.1 microns × 50 mm, 1.8 µm; 1200 bar maximal pressure; Agilent, Santa Clara, CA, USA). In optimized conditions the mobile phase consisted in solvent A (0.05% trifluoroacetic acid) and solvent B (acetonitrile:methanol 70:30 with 0.05% trifluoroacetic acid) using the following gradient: 0 min 35% B. 2 min 35% B, 2.3 min 36.4% B, 3 min 37% B, 4 min 40% B, 5 min 65% B, 6 min 65% B at a constant flow rate of 0.4 mL/min. Unless otherwise stated, injection volume was 1 µL.

Multiple reaction monitoring (MRM) analysis mode was used to monitor the transitions from precursor ions to dominant product ions. Several specific transitions were used to determine each compound, and the most intense transition was used for the quantitation (quantifier transition). The optimized source parameters were: capilar voltage 3 kV, gas curtain temperature 300 °C, gas flow 12 L/min, cell acceleration voltage 2 V, a nebulizer (40 psi). For each transition, the collision energy applied was optimized in order to detect the greatest possible intensity. Dwell time 20 ms. Data acquisition was performed with the software Mass Hunter Workstation Quantitative Analysis version B06 SP01 (Agilent, Santa Clara, CA, USA).

#### 4.1.4. Calibration Curves and Lower Limit of Quantitation

Method validation was performed by studying the linear dynamic range, precision of the analysis, and limit of detection (LOD) and quantitation (LOQ) for the standard compounds. The linear dynamic range was evaluated using serial dilutions of standard stock solutions and plotting the peak area of the quantifier transition of each stilbene vs. the nominal concentration of each corresponding standard compound. The upper linear response was determined by fitting the data starting at a moderately low concentration (from 0.2 to 1.6 mg/L depending on the compound) to a straight line by linear regression analysis method. High concentration data were removed until R^2^ ≥ 0.99. The LODs and LOQs were established for each compound as the concentration of standard providing a signal to noise ratio ≥5 and ≥10, respectively. For the construction of calibration curves of individual stilbenes, serial dilutions from the standard mix solution were performed. The volume injected is the same as the one mentioned above.

### 4.2. Method Applications

#### 4.2.1. Analysis and Quantitation of Stilbenes Produced by *V. vinifera* Cell Culture Upon Elicitation

*Vitis vinifera* cv. Gamay cell cultures were handled and subjected to elicitation as described in [2]. Samples of stilbenes from the extracellular medium and from cells were prepared as described in [5]. One µL aliquot was injected for LC-MS MRM analysis.

#### 4.2.2. Biotransformation Assays

Crude protein extracts were prepared from elicited *V. vinifera* cv. Gamay cell cultures as described in [6]. Resveratrol hydroxylation reaction was assayed as described in [39,53]. The reaction mixture contained 0.2 mM of *trans*-R delivered in DMSO (Fluka Chemika-Sigma Aldrich, St. Louis, MO, USA), 1 mM ascorbic acid, 1 mM NADPH, and 100 mM potassium phosphate buffer, pH 7.4, and started by adding 50 µL of protein extract to complete a final volume of 1 mL. The reaction mixture was incubated for 1 h at 37 °C in the dark and terminated by the addition of 0.5 mL ethyl acetate to extract stilbenoids twice. The solvent was evaporated in a Speed-vac (Eppendorff, Hamburg, Germany) and the solid residue resuspended in 0.2 mL 80% methanol. One µL of reaction medium extract was injected for UHPLC-MS MRM analysis under the optimized conditions.

Absolute concentration of stilbenes in real samples of cell extracts and enzymatic reaction extract was determined using an external calibration curve performed with three levels of standards in duplicate.

#### 4.2.3. Analysis and Quantitation of Stilbenes in Red Wine

Red wine (80% Cabernet Sauvignon and 20% Alicante Bouschet), used in this study was elaborated during the 2012 vintage in a local wine cellar and the analyzed sample was taken right after the alcoholic fermentation and then clarified by centrifugation at 10,000× *g* for 20 min. Wine samples of 2 mL were extracted by solid phase according to *Kallithraka* et al. [40]. After that, extracted stilbenes were evaporated at 30 °C in a Speed-Vac centrifuge (Eppendorff, Hamburg, Germany), resuspended in 60 µL methanol 80% and stored at −20 °C until analysis. One µL of sample extract was injected for UHPLC-MS MRM analysis under the optimized conditions. To minimize the effects of sample preparation on analysis, a sample of wine was spiked with the standard mix, four serial dilutions were prepared by mixing the spiked sample with fresh wine before proceeding to solid phase extraction. A non-spiked wine aliquot was extracted in parallel. Then, concentration of the standard added to the wine sample was plotted against the measured peak area of the quantifier transition of the extracted sample. In this way the percentage of stilbene recovery from wine was obviated and the real concentration of each stilbene compound is obtained from the intercept of the fitting straight line with the standard concentration axis (A = Ao + F.C, where A is the peak area of any sample; Ao is the peak area of the non-spiked sample, F is the response factor and C standard concentration), as shown in Figure S1.

## Figures and Tables

**Table 1:** MRM parameters, chromatographic attributes, and quantitative response of stilbene compounds in standard samples.

Compound	Formula	Mass (Da)	Precursor Ion (*m*/*z*)	Product Ion (*m*/*z*)	Collision Energy (eV)	% Intensity	Retention Time (Min–Max) *trans*/*cis*	LOD (mg/L)	LOQ (mg/L)	Linearity ^b^
**Piceid**	C_20_H_22_O_8_	390.13	391.1	114.8	20	86.5	0.93–0.99/1.52–1.63	0.04	0.07	640
				308.8	8	67.5				
				349.9	0	100				
				229.1 ^a^	8	24.4				
**Resveratrol**	C_14_H_12_O_3_	228.08	229.09	107.1 ^a^	24	100	1.85–1.99/2.55–2.65	0.12	0.22	800
				91 ^a^	24	45.7				
				135 ^a^	8	80.9				
				165 ^a^	28	35.6				
**Piceatannol**	C_14_H_12_O_4_	244.07	245.08	107.1	20	95.5	1.25–1.35/1.90–2.00	0.12	0.15	1600
				135.1	12	100				
				152	36	11.6				
				181.1	24	38.7				
**ε-Viniferin**	C_28_H_22_O_6_	454.14	455.15	107.1	32	100	3.00–3.17/2.80–2.90	0.07	0.09	1777
				215.1	20	75.0				
				349.1	16	38.5				
				199.1	24	32.0				
**Pteroestilbene**	C_16_H_16_O_3_	256,11	257.12	181	40	90.1	5.18–5.28/5.26–5.35	0.06	0.08	1000
				133.1	12	100				
				91	28	58.5				
				165.1	40	72.2				

^a^ Transitions used for piceid analysis. ^b^ Ratio between the highest and lowest concentrations in linear range.

**Table 2:** Quantitation of stilbenes in *V. vinifera* cell culture extracts upon elicitation.

Compound	Concentration (mg/L) in		
	Extract #1	Extract #2	Extract #3
**Piceid**	10.92	14.07	16.35
**Resveratrol**	46.93	62.24	50.82
**Piceatannol**	1.44	1.46	1.38
**ε-Viniferin**	1.48	0.93	0.11
**Pteroestilbene**	n.d.	n.d.	n.d.

**Table 3:** Quantitation of stilbenes in red wine extract.

	t-Piceid (mg/L)	t-Resveratrol (mg/L)	t-Piceatannol (mg/L)	ε-Viniferin (mg/L)	t-Pterostilbene (mg/L)
wine	0.826	0.281	n.d.	0.014	n.d.
S/N	140.24	60.86	0.93	15.6	1.67

n.d. Not detected; S/N Signal-to-Noise.

## Supplementary Materials

The supplementary materials are available online.
