# Supplementary material for: A Focused Multiple Reaction Monitoring (MRM) Quantitative Method for Bioactive Grapevine Stilbenes by Ultra-High-Performance Liquid Chromatography Coupled to Triple-Quadrupole Mass Spectrometry (UHPLC-QqQ)
Source: Molecules. 2017 Mar 7;22(3):418. doi: 10.3390/molecules22030418 (PMC6155428; doi:10.3390/molecules22030418)
Supplement: Supplementary file 1 [file molecules-22-00418-s001.zip › molecules-177789-supplementary/Material supplementary fileS2 (english edits).docx]

Supplementary Tables and Figures

**Table S1.**Quantitation of piceid in standard mixture.

| PICEID | | | |
| --- | --- | --- | --- |
| *Retention time (min)* | ***Nominal concentration (mg/L)*** | ***Calculated concentration (mg/L)*** | ***Standard  deviation*** |
| 0.93 | 0.0625 | 0.048 | 0.004 |
| 0.94 | 0.125 | 0.141 | 0.008 |
| 0.94 | 0.25 | 0.273 | 0.0104 |
| 0.94 | 0.5 | 0.587 | 0.0093 |
| 0.90 | 0.8 | 0.983 | 0.0039 |
| 0.90 | 4 | 3.91 | 0.024 |
| 0.90 | 8 | 7.395 | 0.0048 |
| 0.91 | 40 | 41.075 | 0.225 |

**Table S2.** Quantitation of resveratrol in standard mixture.

| RESVERATROL | | | |
| --- | --- | --- | --- |
| *Retention time (min)* | ***Nominal concentration  (mg/L)*** | ***Calculated concentration (mg/L)*** | ***Standard deviation*** |
| 1.86 | 0.0625 | 0.058 | 0.005 |
| 1.87 | 0.125 | 0.125 | 0.012 |
| 1.85 | 0.2 | 0.348 | 0.005 |
| 1.84 | 0.4 | 0.525 | 0.023 |
| 1.84 | 2 | 1.991 | 0.089 |
| 1.84 | 4 | 3.678 | 0.006 |
| 1.85 | 10 | 10.039 | 0.502 |
| 1.85 | 50 | 50.016 | 0.101 |

**Table S3.** Quantitation of piceatannol in standard mixture.

| PICEATANNOL | | | |
| --- | --- | --- | --- |
| *Retention time  (min)* | ***Nominal concentration (mg/L)*** | ***Calculated concentration (mg/L)*** | ***Standard deviation*** |
| 1.24 | 0.09 | 0.082 | 0.004 |
| 1.24 | 0.14 | 0.122 | 0.008 |
| 1.26 | 0.225 | 0.240 | 0.009 |
| 1.20 | 0.32 | 0.322 | 0.009 |
| 1.20 | 1.6 | 1.65 | 0.020 |
| 1.20 | 3.2 | 3.211 | 0.005 |
| 1.205 | 16 | 15.690 | 0.518 |
| 1.20 | 32 | 30.844 | 0.185 |
| 1.20 | 80 | 74.213 | 0.086 |
| 1.199 | 160 | 167.125 | 0.910 |

**Table S4.** Quantitation of viniferin in standard mixture.

| ε-VINIFERIN | | | |
| --- | --- | --- | --- |
| *Retention time  (min)* | ***Nominal concentration (mg/L)*** | ***Calculated concentration (mg/L)*** | ***Standard deviation*** |
| 3.013 | 0.1125 | 0.118 | 0.0061 |
| 3.001 | 0.5625 | 0.599 | 0.0170 |
| 3.001 | 0.125 | 1.316 | 0.0157 |
| 2.954 | 1.6 | 1.246 | 0.0251 |
| 2.94 | 8 | 9.967 | 0.2573 |
| 2.95 | 80 | 80.901 | 1.876 |
| 2.95 | 160 | 159.534 | 2.739 |

**Table S5.** Quantitation of pterostiblene in standard mixture.

| PTEROSTILBENE | | | |
| --- | --- | --- | --- |
| *Retention time  (min)* | ***Nominal concentration (mg/L)*** | ***Calculated concentration  (mg/L)*** | ***Standard  desviation*** |
| 5.20 | 0.04 | 0.044 | 0.009 |
| 5.21 | 0.05 | 0.0577 | 0.0170 |
| 5.22 | 0.1 | 0.0974 | 0.0181 |
| 5.22 | 0.25 | 0.241 | 0.00329 |
| 5.22 | 0.5 | 0.555 | 0.00235 |
| 5.20 | 0.8 | 1.075 | 0.0759 |
| 5.22 | 4 | 4.230 | 0.162 |
| 5.14 | 8 | 8.629 | 0.0508 |
| 5.14 | 40 | 37.723 | 1.947 |

**Figure S1.** Plot of standard concentration added vs peak area of the quantifier transition for piceid, resveratrol, and ε-viniferin in red wine extract.


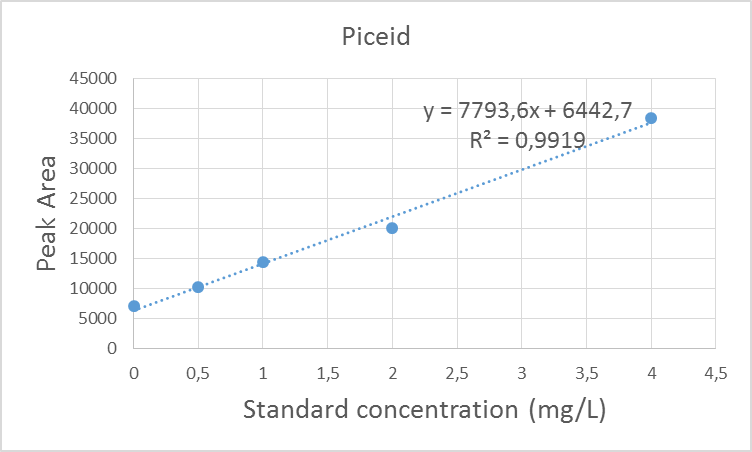

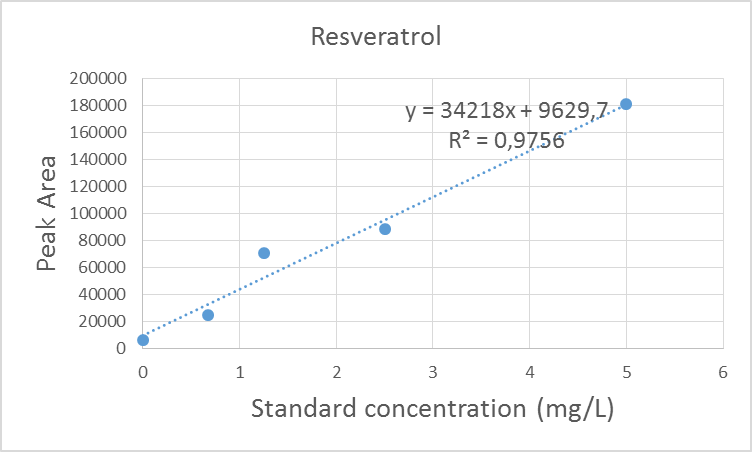

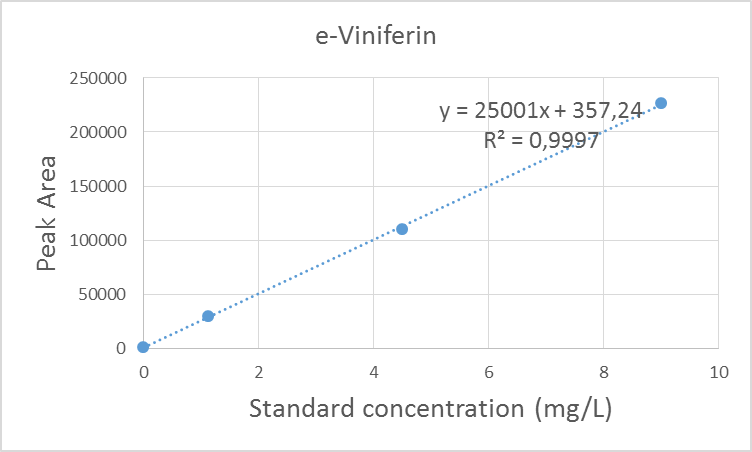


**Figure S2.** Chromatogram HPLC-UV of red wine extract.

UV detection wavelength 306 nm.
